# Medicated thread moxibustion for alopecia areata: A case report

**DOI:** 10.1097/MD.0000000000017793

**Published:** 2019-11-01

**Authors:** Yi-Mei Zhang, Cui-Hong Liu, Yong-Cheng Wang, Hong-Li Teng, Xian-Liang Meng, Xing-Jun Han

**Affiliations:** a Department of Acupuncture and Moxibustion, Second Affiliated Hospital of Shandong University of Traditional Chinese Medicine; b Department of Ultrasound Diagnosis and Treatment, Provincial Hospital Affiliated to Shandong University; c Department of Cardiovascular, Affiliated Hospital of Shandong University of Traditional Chinese Medicine, Jinan; d Department of Toxicology, Guangxi International Zhuang Medical Hospital, Nanning; e Department of Cardiovascular, Taian Hospital of Traditional Chinese Medicine, Taian; f Department of Prevention and Health Centres, Second Affiliated Hospital of Shandong University of Traditional Chinese Medicine, Jinan, China.

**Keywords:** alopecia areata, Kuihua point, medicated thread moxibustion, traditional Zhuang nationality medicine

## Abstract

**Rationale:**

According to the literature reports and clinical studies on alopecia areata (AA) from 2008 to 2018, most clinical treatments have been oral drugs and external ointments. At present, systemic immunosuppressive therapy has been widely used in AA, but there are various side effects such as elevated liver enzymes, gastrointestinal discomfort, poor drug compliance, and repeated illness. We present a case report describing a traditional medicine treatment for AA that uses an ethnic therapy of Zhuang medicine, a kind of Traditional Chinese Medicine, namely, medicated thread moxibustion.

**Patient concerns:**

A 36-year-old man endured AA after going through a family misfortune. Half a year ago, his father passed away suddenly. Since then, he suffered continuous anguish, alcoholism and hair loss, especially in the past 2 months. A coin-shaped area of hair loss began to appear at the top of his head and gradually expanded to the surrounding region.

**Diagnoses:**

A diagnosis of AA was made in the dermatology department of a local hospital.

**Interventions:**

The patient was treated with the medicated thread moxibustion method of Traditional Zhuang Medicine at the Kuihua (special points of Zhuang medicine), Zusanli (ST 36), Xuehai (SP 10), Baihui (DU 20), and Taichong (LR 3) points every other day for 4 weeks.

**Outcomes:**

The area of hair loss showed slight improvement after 1 week of treatment. Only just a sprinkling of wooly hairs, whose color and thickness were similar to those of fine facial hairs, began to emerge sporadically from the follicles; they could be seen only in a bright light. When the patient saw the obvious curative effect, we continued the treatment for 2 weeks with the patient's consent. Three weeks later, the patchy AA area was covered with small cotton-like hairs of different lengths and uneven colors.

**Lessons:**

The medicated thread moxibustion method of Zhuang medicine can be an effective alternative treatment in patients with AA.

## 1 Introduction

Alopecia areata (AA) is a type of nonscarring and immune-mediated disease-causing patchy hair loss with no obvious epidermal changes, which often occurs in the parts of the body covered with hair. According to statistics, the lifetime incidence rate of AA is approximately 1.7%; there is a higher incidence in younger patients, but there is no significant gender difference.^[1,2]^ Although hair loss does not directly threaten the life span, it can cause severe mental burden and social stress because it impairs beauty.^[3]^ Currently, the major methods for treating AA include nonspecific broad immunosuppressant medications given systemically or locally to dampen immune cell attack or contact sensitizers to redirect autoimmune attack.^[4]^ However, while these methods offer effective treatment for AA, they also have negative side effects for patients.^[5]^ Additionally, the prognosis of the disease is unpredictable and highly variable. Furthermore, the etiology and pathological mechanism of AA have not been fully elucidated, which creates obstacles to effective treatment strategies and leads to huge medical needs.^[6]^ Here, we report a male patient who presented with spontaneous, progressive hair loss lasting 6 months; the patient achieved very good clinical benefits after being treated with the medicated thread moxibustion method of Zhuang medicine.

## 2 Case report

A 36-year-old male visited our department on August 13, 2018 with a main complaint of loss of hair loss on the top of the head with insomnia, depression, anxiety, and a series of related symptoms after his father died suddenly approximately half a year prior. The clinical examination showed a nonscarring alopecia plaque, which was approximately 5.0 × 3.2 cm in size; there was no involvement beyond the top of the head. The damaged part of the hair loss area had a bright skin color and a clear boundary with its surroundings. By asking for detailed information about his illness, we learned that the patient had no family history or other related illnesses, although the patient did have occasional irregular habits and alcoholism in the 6 months prior. To further clarify the etiology, routine examinations, including thyroid color Doppler ultrasound detection, and a full blood count, were performed to exclude other primary diseases; all results were normal. Based on the above information, the patient refused to accept any medication, including oral or external options. Therefore, we tried to treat the patient with the medicated thread moxibustion method of traditional Zhuang medicine.

**Figure 1:**
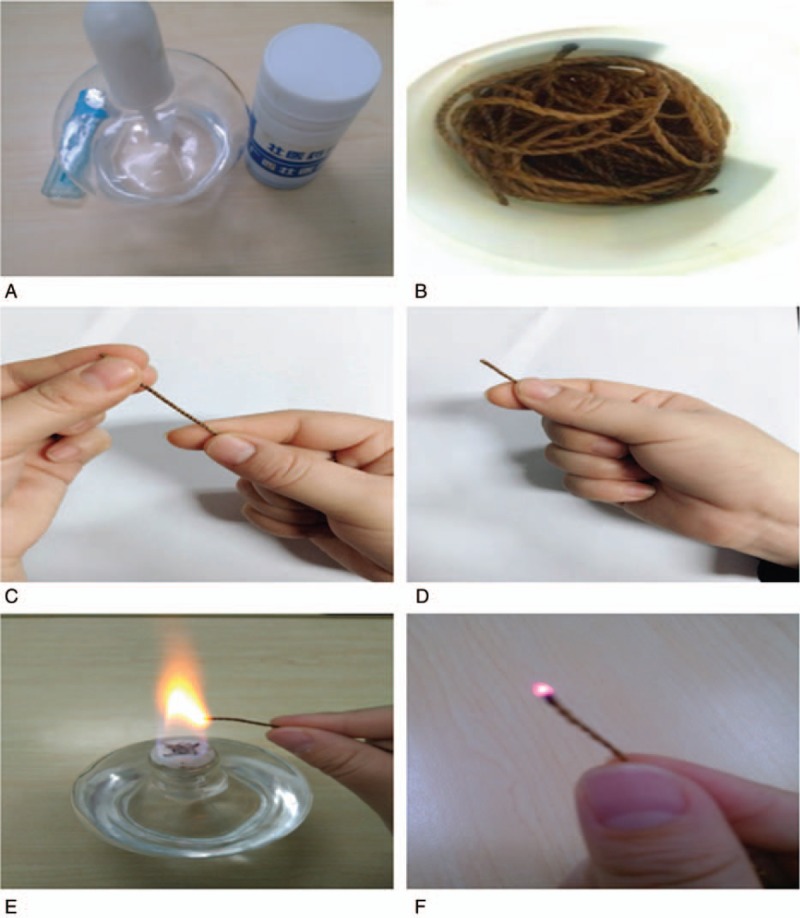
Method and material of medicated thread moxibustion in Zhuang medicine (A–F).

After full communication with the patient, the patient signed an informed consent form for the publication of the case. We chose to use the version of II twine with a diameter of 0.7 mm (the medicated threads were provided by Guangxi International Zhuang Medical Hospital), a lighter and an alcohol lamp (Fig. 1A and B).^[7]^ The first affected skin area is often chosen as the primary acupoint. Here, we chose acupoints including the Kuihua acupoint (dependent on the shape and size of local skin lesions on the body surface, a group of acupoints were selected along the periphery and midpoint, which were sunflower-shaped), Zusanli (ST 36), Xuehai (SP 10), Baihui (DU 20), and Taichong (LR 3). The procedure included the following 4 main steps.

(1) Arranging: the loose thread was tightly wound (Fig. 1C).
(2) Holding: the end of a thread was grabbed with the forefinger and thumb of the right hand 1 to 2 cm were exposed (Fig. 1D).
(3) Igniting: the exposed end was lit with an alcohol lamp. If there was a flame, it was extinguished; only a bead-like sparkle was needed on the top of the medicated thread (Fig. 1E).
(4) Moxibustion: the acupoints were aligned with the bead-like sparkle on the thread; the thumb and index finger gripped the end of the thread in a steady and agile manner with wrist and thumb flexion movements (Fig. 1F).

When the sparkle was extinguished, the thread was picked up immediately; this called 1 Zhuang. Repeating this sequence of steps, moxibustion was performed at the above 5 acupoints in turn. Each acupoint was cauterized 2 times, once every 2 days for 4 weeks. After moxibustion on the day of treatment, the patients were told to not scratch or wash their heads with water. Additionally, the patient was instructed to stay relaxed throughout the treatment period and to participate in the follow-up for 3 months.

**Figure 2:**
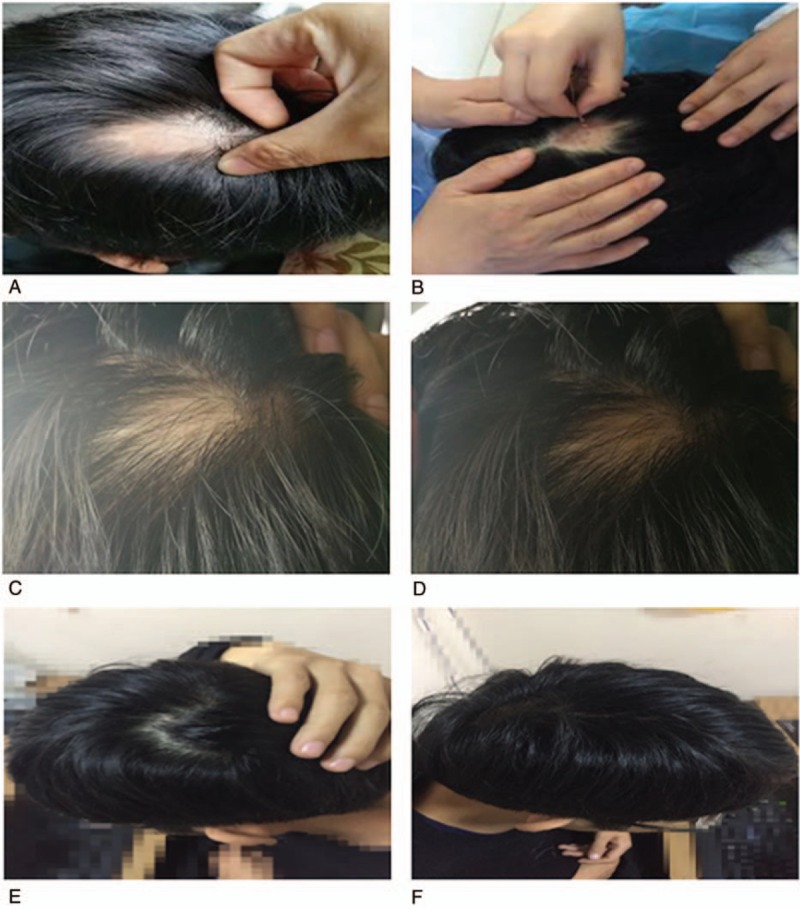
Patchy hair loss affecting the vertex 5.0 × 3.2 cm. This picture was taken during the first treatment (A). The performance of Zhuang medicine medicated thread moxibustion of at the patient's Kuihua acupoint on his head in the first week (B). The second week of Zhuang medicine medicated thread moxibustion treatment (C). The third week of Zhuang medicine medicated thread moxibustion treatment (D). Fourth week of Zhuang medicine medicated thread moxibustion treatment (E). The follow-up period was 3 months after the end of treatment (F).

After the initial treatment, the area of hair loss did not continue to expand, and no new areas of hair loss mass appeared (Fig. 2A). After 1 week of treatment, a small amount of hair began to appear sporadically from the hair follicles in the hair loss area. The color and thickness of the hair were similar to those of fine hair, and the hairs could only be seen in bright light (Fig. 2B). After the patient received medicated thread moxibustion for 2 weeks, hairs of different lengths and uneven colors were observed to cover the patchy AA area (Fig. 2C). After 3 weeks of topical treatment with medicated thread moxibustion, the patchy AA area was covered with hair of different lengths and colors (Fig. 2D). At the end of the last course of treatment, the hair follicles in the hair loss area were almost covered with hair. Although the density, thickness, and color were not exactly the same as the surrounding hair, the hair was still in the process of regeneration (Fig. 2E). After the medicated thread moxibustion treatment, we stopped moxibustion and followed the patient closely for 3 months (Fig. 2F). Additionally, a hair pulling procedure was conducted on newly grown hair at the end of each course and during the follow-up period, and the results were negative. Informed written consent was obtained from the patient for publication of this case report and accompanying images. All procedures involving the patient were conducted in accordance with the ethical standards of the Ethics Committee of the Second Affiliated Hospital of Shandong University of Chinese Medicine.

## 3 Discussion

AA is an autoimmune disease characterized by an infiltration of T cells and the production of inflammatory factors, the most typical manifestation of which is a sudden exfoliation of scalp plaques.^[8]^ Studies have shown that T-helper 17 cells (Th17) and regulatory T cells (Tregs) play important roles in the pathogenesis of autoimmune diseases.^[9]^ Th17 cells can promote inflammation by releasing cytokines, while an increase in Treg cells can limit the activity of inflammatory factors.^[10]^ Th17 cells are a unique subset of T-helper cells that can produce cytokines such as interleukin (IL)-17A, IL-17F, IL-21, IL-22, IL-6, and tumor necrosis factor-a (TNF-a).^[11–14]^ Relevant research shows that IL-17, TNF-a, transforming growth factor-β (TGF-β) and other pro-inflammatory factors are significantly increased in the pathogenesis of AA. Atwa et al. found that IL-17 and TNF-a were positively correlated with the severity of AA, while the IL-22 level was positively correlated with the duration of AA.^[15]^ Recent studies have also shown that there is a dense infiltration of IL-17 cells around hair follicles and dermal stromal areas in AA patients.^[16,17]^ In vitro studies have shown that TNF-a can induce the vacuolation of stromal cells, abnormal keratosis of follicular balloons and inhibition of hair growth, while IL-6 can enhance the differentiation of Th17 cells by promoting the continuous junction of the IL-21/IL-23 pathway.^[18,19]^ Therefore, it can be concluded that the related inflammatory factors secreted by Th17 cells may be an important factor in AA induction.

Increasing evidence shows that Treg cells are important cellular components for maintaining immune self-tolerance and homeostasis, and abnormalities of these cells may lead to autoimmune and immunopathological diseases.^[20]^ Tregs are a special subset of CD4^+^ T cells that prevent autoimmune diseases by secreting soluble factors such as TGF-β and IL-10.^[21]^ Researchers have pointed out that the collapse of immune privileges of hair bulbs during growth due to the impairment of Treg or TGF-β function is the key mechanism leading to AA.^[22]^ Zoller et al found that the CD4^+^/CD25^+^ Treg level was significantly decreased in a C3H/HeJ AA mouse model.^[23]^ Tembhre et al found that the serum level of TGF-β1 was decreased in AA patients, suggesting that Treg function was deficient.^[24]^ Consequently, the T cell-mediated immune enhancement and Treg deficiency-induced decline in immune tolerance are important pathogenesis of AA.

Medicated thread moxibustion is a traditional national therapy that uses a variety of Zhuang medicine methods involving the preparation of special ramie thread that has been soaked in liquid and direct moxibustion of the corresponding acupoints or lesions of the human body after ignition. Medicated thread moxibustion has unique thermal and concentrated thermal radiation effects and involves the release of various drug molecules during combustion; these molecules are quickly adsorbed on the human body surface after high-temperature fumigation, forming a high drug concentration area around the epidermis, and are quickly transported to the whole body through the meridian system. Relevant experimental studies showed that medicinal thread moxibustion upregulated serum IgG and C3 levels and downregulated IgE levels in asthmatic patients with lung deficiency,^[25]^ increased the levels of CD3, CD4, and CD4/CD8 in the peripheral blood of patients with herpes zoster,^[26]^ and downregulated the expression of IL-17F in ulcerative colitis patients.^[27]^ Therefore, in this study, the mechanism of medicated thread moxibustion in the treatment of AA may be related to the regulation of Th17 or Treg cells, and relevant validation will be implemented in future experiments.

## Author contributions

**Conceptualization:** Yi-Mei Zhang, Cui-Hong Liu, Yong-Cheng Wang, Xing-Jun Han.

**Data curation:** Yi-Mei Zhang, Cui-Hong Liu, Yong-Cheng Wang, Hong-Li Teng, Xian-Liang Meng.

**Formal analysis:** Yi-Mei Zhang, Cui-Hong Liu, Yong-Cheng Wang.

**Funding acquisition:** Hong-li Teng.

**Investigation:** Yi-Mei Zhang, Yong-Cheng Wang, Hong-Li Teng, Xian-Liang Meng.

**Methodology:** Yi-Mei Zhang, Cui-Hong Liu, Yong-Cheng Wang, Hong-Li Teng, Xian-Liang Meng.

**Resources:** Yi-Mei Zhang, Cui-Hong Liu, Yong-Cheng Wang, Hong-Li Teng, Xian-Liang Meng, Xing-Jun Han.

**Software:** Yi-Mei Zhang, Cui-Hong Liu, Yong-Cheng Wang, Xian-Liang Meng.

**Supervision:** Xing-Jun Han.

**Validation:** Hong-li Teng, Xing-Jun Han.

**Visualization:** Yi-Mei Zhang, Cui-Hong Liu, Yong-Cheng Wang, Xing-Jun Han.

**Writing – original draft:** Yi-Mei Zhang, Cui-Hong Liu, Yong-Cheng Wang.

**Writing – review and editing:** Yi-Mei Zhang, Cui-Hong Liu, Yong-Cheng Wang, Xing-Jun Han.

xing-jun Han orcid: 0000-0001-7343-4385.
